# Sustainable Growth Drivers: Unveiling the Role Played by Carbon Productivity

**DOI:** 10.3390/ijerph19031374

**Published:** 2022-01-26

**Authors:** Wenhao Qi, Changxing Song, Meng Sun, Liguo Wang, Youcheng Han

**Affiliations:** 1 School of Economics and Management, Jilin Agricultural University, Changchun 130118, China; hao2005buct@jlau.edu.cn (W.Q.); songchangxing@jlau.edu.cn (C.S.); 1818300024@e.gzhu.edu.cn (Y.H.); 2 Center for Northeast Asian Studies, Jilin University, Changchun 130012, China; 3 School of Finance, Jilin University of Finance and Economics, Changchun 130117, China; wlg@jlufe.edu.cn

**Keywords:** carbon productivity, growth drivers, convergence mechanism, spatial effects, environmental protection

## Abstract

In global climate change, improving carbon productivity holds great importance for China’s sustainable growth. Based on panel data of 30 Chinese provinces and cities from 1997–2017, the drivers, spatial effects, and convergence characteristics of carbon productivity in China are explored by combining a factor decomposition framework and a spatial panel model. The findings show that (1) China’s carbon productivity shows continuous positive growth, and the substitution effect of capital for energy dominates this changing pattern; (2) There is a β-convergence trend and club convergence in China’s carbon productivity, and the spatial technology spillover accelerates the convergence rate; (3) With its accelerated industrial transformation and technological upgrading, China’s current carbon productivity converges faster than its earlier stage, and the role of physical capital investment has gradually shifted to suppression. In contrast, the positive push of human capital investment has been strengthened; (4) From the perspective of the realization mechanism, the convergence of carbon productivity in China mainly comes from the convergence of energy restructuring and capital-energy substitution. These findings can help China narrow the inter-provincial carbon productivity gap in terms of improving factor structure, upgrading technology, etc., and provide references for sustainable growth decision making in China and around the world.

## 1. Introduction

In recent years, climate change issues such as extreme weather and glacier retreat have received widespread attention from academic and political circles. Climate change and its impacts have become one of the world’s most severe environmental problems [1]. The large number of greenhouse gases emitted by human economic activities is the leading cause of the global slowdown and ecological degradation. Carbon dioxide is the most critical greenhouse gas [2,3]; governments have put forward low-carbon action plans to meet the challenge, thus driving [4] both macroregions [5] and microenterprises to actively explore sustainable development measures. However, the UN Emissions Gap Report 2021 states that countries’ current autonomous action contributions are insufficient to meet the commitments of the Paris Agreement. The global temperature will still rise by a catastrophic 2.7 °C, which will cause irreversible damage to the global climate, and countries must accelerate emissions reductions to keep temperature increases below 1.5 °C [6]. The recent success of the 26th Conference of the Parties to the United Nations Framework Convention on Climate Change (COP26) resulted in unprecedented commitments to protect forests, set “net zero emissions targets,” reduce methane emissions, and accelerate green technologies. For example, the UK supports sustainable infrastructure and revolutionary green technologies in developing countries. France has proposed climate finance support for developing countries, and Japan has joined a global agreement to cut methane emissions.

Since its reform and opening-up, China’s economy has maintained rapid double-digit growth for a long period of time, providing Chinese experience and solutions for developing countries. Environmental pollution and ecological imbalance inevitably emerged during the industrialization process in developed Western countries, and China has now surpassed the USA as the largest carbon emitter. China is actively participating, leading, and contributing to global climate change prevention and control to assume its significant power to reduce emissions and strengthen climate change action [7]. As the largest developing country, the income level of Chinese residents is still far from that of developed countries, and the choice of intensity emission reduction is in line with the development vision of shared prosperity for its citizens. Before COP15 in Copenhagen, the Chinese government announced a 40–45% reduction in carbon dioxide emissions per unit of GDP (carbon intensity) by 2020 compared to 2005. In 2020, at the Climate Ambition Summit, President Xi Jinping further proposed a voluntary reduction target of more than 65% of CO_2_ emissions per unit of GDP by 2030 compared to 2005. By the end of 2020, China’s carbon intensity had fallen by 48.4% compared to 2005, exceeding the autonomous commitment target and reducing cumulative CO_2_ emissions by about 5.8 billion tons, basically reversing the rapid growth of CO_2_ emissions [8]. It is now widely believed that sustainable development cannot be achieved by increasing factor inputs alone. If it reaches peak emissions by 2030, China will face enormous pressure to reduce emissions.

Carbon Productivity (CP) is a core concept in evaluating low-carbon economic development [9] and is another core indicator of sustainable economic growth, after labor productivity and capital productivity. The McKinsey Global Institute report *The Carbon Productivity Challenge: Stemming Global Change, Sustaining Economic Growth* states that successful climate change action must support the goals of stabilizing greenhouse gases and sustaining economic growth, i.e., there must be a significant increase in total GDP output per unit of carbon equivalent emissions. Therefore, improving carbon productivity is an effective way to achieve coherence between emissions reductions and economic growth [10,11], and thus an essential basis for governments to formulate long-term sustainable development policies. For a long time, research on economic growth has focused on labor productivity and capital productivity, while less attention has been paid to carbon productivity for sustainable development. Will carbon productivity eventually increase with economic growth? What are the essential factors that drive carbon productivity improvements? Will carbon-productivity differences between regions eventually converge? Suppose lower-carbon-productivity regions do not spontaneously catch up with developed areas. In that case, this will not only undermine the achievement of overall national sustainable development goals, but the significant economic disparities could also lead to a series of serious social problems, such as increased energy poverty in household welfare [12]. Therefore, a solution to the above issues is vital for stabilizing growth and promoting emission reduction. Studying the drivers of carbon productivity and the convergence characteristics in China provides new cases and lessons for other developing countries in the world.

The environmental Kuznets curve (EKC) literature provides a starting point for this paper, as empirical evidence suggests that the pattern of change between carbon productivity and economic growth arises from the influence of different drivers [13,14]. Within the sustainable growth framework, data envelopment analysis (DEA) is widely used to better reveal the relationship between drivers and output growth [15]. Evidence of economic convergence and the spatial panel data approach suggests the possibility of carbon productivity convergence and the significant effect of spatial dependence [9,16]. Based on the evidence from the EKC literature, this leads to the research question of this paper. We attempt to provide a new analytical framework and Chinese experience for sustainable growth decisions under global energy conservation and emission reduction by integrating factor decomposition techniques and convergence analysis methods to study the carbon productivity problem in China. Therefore, this paper aims to achieve the following three objectives: (1) to integrate a comprehensive decomposition framework to account for the growth of the main drivers of carbon productivity in China; (2) to construct a carbon productivity convergence equation based on economic growth theory to examine the convergence characteristics and driving mechanisms of regional carbon productivity in China; and (3) to introduce geographical factors into the convergence equation to explore the spatial spillover effects on regional carbon productivity convergence. The follow-up structure of this study first presents a literature review to track the latest research progress on related issues. Second, a normative research framework is proposed to explain the research methodology and data situation. Again, the study results are presented in layers and fully discussed according to the empirical specification. Finally, the study reports the main conclusions, policy recommendations, and outlook for future research.

## 2. Literature Review

### 2.1. Carbon Productivity and Economic Growth

The existing literature does not provide direct evidence of the relationship between carbon productivity and economic growth. Since carbon productivity is precisely the ratio of output to emissions, a logical starting point for this paper can be established from the literature that explores the relationship between carbon emissions and economic growth. Therefore, the literature on the relationship between carbon productivity and economic growth can be traced back to the study of the EKC hypothesis [17]. The EKC hypothesis supports the inverted U-shaped relationship between economic growth and CO_2_ emissions [18]. Still, some evidence also suggests a linear relationship between the two, and suggests that the positive effects of structural changes in the economy and technological progress on emission levels persist [19]. Since there is a certain inverse correspondence between carbon emissions and carbon productivity, it is easy to see from the differences in studies in the early literature that the pattern of time variation in the relationship between carbon productivity and economic growth is not certain.

In a recent study, an empirical test based on a larger sample provided strong evidence for the EKC hypothesis of the relationship between carbon emissions and economic growth, by estimating a panel smooth transition regression model for 40 countries over the period of 1994–2016 [20]. By using a simplified form of the application model, this literature supported the EKC hypothesis for developed economies [21], upper-middle-income economies [22], and emerging economies [23]. In addition, there is also literature that validated the N-shaped curve hypothesis and identified a more complex nonlinear relationship between carbon emissions and economic growth [24]. From this perspective, the latest study also further described that the EKC hypothesis was not rejected [25,26]. However, a study based on time series data at the individual country level provided little evidence for the EKC hypothesis for the Arctic countries [27].

The available studies more and more strongly support a nonlinear relationship between carbon emissions and economic growth. Due to the advantages of energy structure [13,28], financial markets [20,29], and technology levels [14,30], the evidence of decoupling between the two mainly comes from countries with higher income levels [31]. This also implies that the inverted U-shaped relationship between carbon emissions and economic growth estimated by panel data is not necessarily applicable to individual countries, as the development characteristics of low-income countries on the left of the inflection point cannot be effectively captured, which further strengthens the necessity of additional research on similar characteristics and even specific countries or regions [32]. Therefore, the identification of complex time-varying patterns may be further facilitated by decomposition accounting for carbon emission drivers. If there is an inverted U-shaped relationship between carbon emissions and economic growth, then carbon productivity will show a pattern of slow growth followed by rapid growth in change. A more complex nonlinear relationship reveals a reconnected form of carbon productivity and economic development. In this paper, the following research hypothesis is formulated using carbon productivity as the subject of study, in conjunction with the empirical evidence provided by the above literature.

**Hypothesis** **1** **(H1).**  *China’s carbon productivity growth exhibits a nonlinear pattern of change.*

### 2.2. Drivers of Carbon Productivity

The existing literature most often uses parametric regression and nonparametric decomposition to identify drivers of carbon productivity.

Parametric regressions are mainly based on parametric tests of econometric models, which analyze the impact of relevant factors on carbon productivity. Numerous studies have shown that industrial development’s effects on carbon productivity are heterogeneous and do not behave precisely in different countries or regions. For example, the concrete industry in Canada [33], the metallurgical industry in China [34], and industrial development in Pakistan [35] are inefficient, and the extensive growth of these industries hurts carbon productivity improvement. In China’s Yangtze River Delta region, industrial development was efficient, and industrial growth facilitated provincial carbon productivity improvements [36]. In addition, previous studies supported the EKC hypothesis of the relationship between urbanization and carbon productivity in developing countries. They suggested that urbanization in developing countries was still decreasing carbon productivity and increasing carbon emissions [37]. However, a recent study indicated that urbanization in China has brought about concentrated energy use and technological advances, thereby increasing carbon productivity while decreasing carbon emissions [38]. Admittedly, any increase in GHG emissions will affect the development of technologies aimed at GHG reduction [39]. Therefore, technological progress becomes a significant factor in reducing energy intensity [40,41]. With the widespread use of spatial measurement techniques, a growing body of literature has intensified the examination of spillover effects in analyzing factors influencing carbon productivity. A recent study using spatial panel data for 17 Chinese provinces showed that the slow increase in carbon productivity had a significant spatial spillover effect. The patterns of industrial development and urbanization affecting carbon productivity were homogeneous and mimicked each other across the 17 provinces studied [42]. Another similar study used the panel data of 30 provinces in China to identify the multiple effects of GDP, urbanization rate, industrial structure, energy structure, energy intensity, technological innovation, openness, and foreign direct investment on carbon productivity. It argued that economic and energy-related emission reduction measures were still the key to achieving Chinese provinces’ carbon intensity emission reduction targets [43].

Nonparametric methods often use exponential decomposition analysis (IDA) to separate the drivers of carbon productivity. Among them, the log-averaged Divisia index (LMDI) method is the most widely used in practice, for its advantages of leaving no residuals, simplifying interpretation, and consistency of decomposition formulas [44]. The LMDI method was used for a study on the manufacturing industry in Latvia to decompose the change in total CO_2_ emissions into five different factor effects: industrial activity, structural change, energy intensity, fuel mix, and emission intensity. Further analysis showed that increased energy-intensive industrial activity could largely offset the positive effects of improved energy efficiency and decarbonization measures [45]. The literature combined the LMDI approach with a spectral clustering approach to describe the spatial and temporal differences in industrial CO_2_ emission factors across 30 Chinese provinces. Energy intensity and GDP per capita played a dominant role in suppressing and promoting industrial CO_2_ emissions [46]. By considering the accounting principle of electricity transfer consumption, a study estimated the carbon productivity of the Chinese electricity industry. It decomposed the LMDI of electricity carbon productivity based on regional and industry demand perspectives. The analysis concluded that the environmental and economic efficiency of the electricity industry, although negative, had a more significant impact on scale effects than technology effects on other industries [47] In addition, a recent study further explored the growth potential of carbon productivity across Chinese provinces based on conventional decomposition analysis combined with cluster analysis, and revealed that policymakers should focus on optimizing the industrial structure and reducing energy intensity to promote sustainable development, and fully consider regional heterogeneity [48].

Although the existing literature has made many valuable explorations into the drivers of carbon productivity, the economic mechanisms and policy interface are not sufficiently discussed. A complete accounting framework reveals that the drivers’ contributions are still lacking. The IDA method contains information that factor effects overlap, and it is difficult to provide a reasonable economic explanation for carbon productivity changes [49]. In recent years, nonparametric methods based on DEA have also been more widely used in environmental efficiency evaluation. Carbon emissions are considered input factors [15] or undesired outputs [50] in DEA models, and the optimal solution of the model is usually used to measure carbon efficiency [51]. Based on the multiperiod efficiency indices of different production technology DEA models, total factor productivity (TFP) can also be calculated by constructing productivity indices. It can be easily decomposed into technical efficiency, scale efficiency, and technological progress [52,53]. For the DEA model in the literature with a single-period production technology as the reference, technological progress is not continuously comparable within the time series [54], which can easily lead to misjudging the pattern of productivity change influenced by the drivers. In addition, some DEA literature that introduces carbon emission constraints still refers to efficiency indices as carbon productivity, which is not conducive to the essential distinction between “single-factor” and “full-factor” productivity measures [9]. Although single-factor carbon productivity does not consider the capital, labor, and other factors [55], the input–output principle allows for linkages between factors. Total factor productivity as a driver of single-factor carbon productivity is the focus of this paper. Based on the above analysis, the following research hypotheses are proposed:

**Hypothesis** **2** **(H2).**  *Capital-to-energy substitution dominates the pattern of carbon productivity change in China, and the role of total factor productivity is not sufficient.*

**Hypothesis** **3** **(H3).**  *The asynchrony of capital-to-energy substitution is the main reason for China’s regional differences in carbon productivity.*

### 2.3. Convergence Analysis of Carbon Productivity

A basic consensus is that economic convergence originates from productivity convergence under knowledge spillovers in the study of growth convergence. In academic discourse, neoclassical growth theory assumes that differences in per capita income and capital will disappear as the economic growth rate declines and the economy eventually stabilizes. Endogenous growth theory argues that there are spillover effects of intellectual and human capital accumulation in economic growth, and that low-productivity countries rely mainly on latecomer advantages to catch up with high-productivity countries [56]. In recent years, global warming and energy shortages have received increasing attention, and the evolutionary trends and convergence analysis of carbon emissions and carbon productivity have become a research hotspot. In the context of global emission reduction, countries aim toward economic growth with minimal carbon equivalent consumption, and question whether sustainable development will be stabilized by diminishing marginal returns. Convergence of energy productivity provides the initial empirical evidence that countries or regions with lower initial levels have relatively higher growth rates [57].

The literature on the convergence of sustainable growth can be divided into two lines. The first uses the traditional parametric approach to test for absolute, conditional, and stochastic convergence. The literature that tests the carbon convergence hypothesis using pairwise and club convergence tests shows that club convergence occurs in many countries, while CO_2_ emissions diverge in pairwise tests [58]. Per capita, carbon emissions and carbon intensity show convergence within World Bank member countries, with carbon emissions relatively stable and converging at a lower rate than carbon intensity [59]. Panel data estimates for a sample of Chinese cities also supported the carbon per capita convergence hypothesis but with more remarkable persistence in the towns with lower carbon per capita emissions, greater mobility in the towns with higher carbon per capita emissions, and significant differences in the dynamics of carbon per capita emissions across geographic, income, and environmental policy groups [60]. Panel data tests for a 50-state U.S. sample further validated the conditional convergence of per capita carbon emissions and the existence of multiple club convergences based on industry segmentation [61]. Recent literature examined the impact of spatial effects on sustainable growth convergence. For example, provincial carbon intensity convergence in China is supported by tests of spatial panel data models, where dynamic panel data models converge at a higher rate than cross-sectional regression models, and spatial panel data models converge at a higher rate than nonspatial models [16]. Another study used a similar approach to test the convergence of total factor productivity, including carbon emissions. The results showed that total factor productivity exhibited conditional spatial convergence and club convergence, and that agglomeration externality was essential for increasing carbon productivity and achieving convergence [9]. In addition, some of the literature used nonparametric methods to study the dynamic distribution of sustainable growth. For example, recent literature tested using spatial Markov transfer probability matrices, and found club convergence in the carbon efficiency of Chinese cities [62]. A static test using Fourier functions to assess the convergence of CO_2_ emissions found that emissions are converging in most countries, independent of national development patterns [63].

There is already considerable literature that has explored the environmental convergence hypothesis to some extent. However, further research on carbon productivity convergence is yet to provide additional empirical evidence. Although some of the literature has noted the influence of spatial variation on the convergence characteristics of carbon emissions, the examination of spatial factors of carbon productivity convergence is still missing. On the other hand, technological progress in one economy may be transmitted to other economies, meaning that the closed economy assumption of neoclassical growth theory may be invalid. Moreover, from an econometric perspective, spatial dependence can lead to unreliable statistical inferences if spatial spillover effects exist and are ignored. Based on the above analysis, the following research hypotheses are proposed.

**Hypothesis** **4** **(H4).**  *There are overall convergence and club convergence characteristics of carbon productivity growth in China.*

**Hypothesis** **5** **(H5).**  *The spatial spillover effect further increases carbon productivity’s convergence rate.*

**Hypothesis** **6** **(H6).**  *The drivers play different roles in achieving carbon productivity convergence.*

From a review of the above literature, it is easy to find that the uncertainty in the pattern of carbon productivity change mainly comes from the differential influence of the relevant drivers. These drivers’ differences also determine the mechanism of achieving regional carbon productivity convergence. While exploring carbon productivity changes, drivers, and convergence trends in isolation provides limited evidence for sustainable growth, a convergence factor decomposition and convergence analysis framework appears to provide richer empirical evidence and insights for decision making. Therefore, the following extensions are attempted in this paper to enrich the existing literature: first, construct a DEA decomposition framework that can reflect the growth mechanism by unifying factor substitution, total factor productivity, and single-factor carbon productivity, and then account for the contribution of drivers to carbon productivity improvement. Second, we construct a convergence equation for carbon productivity based on panel data, introduce spatial dependence to test the regional data of China, and then analyze the impact of spillover effects on the rate of convergence and growth potential. Finally, we integrate the power decomposition and convergence equation to further explore the impact of drivers on the intersection of carbon productivity and growth potential.

## 3. Research Methodology and Data Description

### 3.1. Power Decomposition Framework of Carbon Productivity

Carbon productivity is defined as the GDP output per unit of carbon equivalent emissions in a certain period. Since different types of energy consumption can estimate carbon equivalent emissions, and energy consumption per unit of emissions reflects the influence of energy mix, we further isolate the energy mix based on the defining equation, which can be obtained as:

(1) $$CP_{t}=\frac{Y_{t}}{C_{t}}=\frac{E_{t}}{C_{t}}\times \frac{Y_{t}}{E_{t}}=ES_{t}\times YE_{t}$$

In Equation (1), *CP* is carbon productivity, *Y* is GDP, *C* is carbon equivalent emissions, and *E* is fossil energy consumption. *ES* is the energy consumption per unit emission, reflecting the influence of energy structure on carbon productivity; *YE* is the GDP per unit energy consumption output, which is the energy productivity. According to the production theory, energy productivity can form an output relationship with the unit energy factor input. Thus, the effects of factor accumulation and total factor productivity can be further separated. The global DEA model is used in this paper to ensure the continuity and stability of the decomposition of technological progress. *KE* and *LE* denote the capital input and labor input per unit of energy consumption, respectively, and $D_{c}^{g}\;$ is the distance function of output direction under the constant payoff of scale (CRS). Then, the global optimal energy productivity of the production unit in period t is from ${\bar{YE}}_{t}=YE_{t}\times D_{c}^{g}\left(KE_{t},LE_{t},YE_{t}\right)$. Further, the decomposition formula of drivers is obtained as follows:

(2) $$YEC_{t}^{t+1}=\frac{YE_{t+1}}{YE_{t}}=\frac{D_{c}^{g}\left(KE_{t},LE_{t},YE_{t}\right)}{D_{c}^{g}\left(KE_{t+1},LE_{t+1},YE_{t+1}\right)}\times \frac{{\bar{YE}}_{t+1}\left(KE_{t+1},LE_{t+1}\right)}{{\bar{YE}}_{t}\left(KE_{t},LE_{t}\right)}=TFP_{t}^{t+1}\times XEC_{t}^{t+1}$$

In Equation (2), *YEC* and *TFP* denote GDP growth and total factor productivity change per energy consumption unit, respectively. Since the factor input per unit of energy consumption reflects the substitution relationship of capital and labor for energy input, *XEC* is called the factor substitution effect here. According to the principle of DEA model solving, $D_{c}^{g}\;$ is known that the inverse reflects the global technical t  efficiency of a given production unit in the first period. Total factor productivity is thus defined as the ratio of global technical efficiency of two adjacent periods, while the factor substitution effect is defined as the optimal output. Further dynamizing Equation (1) and bringing Equation (2) gives the following decomposition:

(3) $$CPC_{t}^{t+1}=\frac{CP_{t+1}}{CP_{t}}=\frac{ES_{t+1}}{ES_{t}}\times \frac{YE_{t+1}}{YE_{t}}=ESC_{t}^{t+1}\times TFP_{t}^{t+1}\times XEC_{t}^{t+1}$$

*CPC* and *ESC* denote carbon productivity change and energy mix change, respectively. Equation (3) shows that three mechanisms mainly drive carbon productivity growth: first, energy structure optimization, i.e., the reduction in carbon emissions due to the substitution of low-carbon energy for high-carbon energy; second, total factor productivity growth, i.e., the increase in energy production due to the improvement of global technical efficiency in two adjacent periods; third, the factor substitution effect, i.e., the change in global optimal unit productivity due to the substitution of capital and labor for energy under the same technological conditions.

Combining single-period DEA with global DEA, TFP can be further decomposed into technological progress changes and technical efficiency changes. Let $D_{c}^{t}$ be the CRS output distance function of the technical reference set for a single period, thus defining technological progress as the ratio of the relative distances of a given production unit to the single-period frontier and the global frontier, while technical efficiency is still defined as the ratio of the single-period technical efficiencies of the two adjacent periods, calculated as follows:

(4) $$TPC_{t}^{t+1}=\frac{D_{c}^{g}\left(KE_{t},LE_{t},YE_{t}\right)}{D_{c}^{t}\left(KE_{t},LE_{t},YE_{t}\right)}/\frac{D_{c}^{g}\left(KE_{t+1},LE_{t+1},YE_{t+1}\right)}{D_{c}^{t+1}\left(KE_{t+1},LE_{t+1},YE_{t+1}\right)}$$

(5) $$TEC_{t}^{t+1}=\frac{D_{c}^{t}\left(KE_{t},LE_{t},YE_{t}\right)}{D_{c}^{t+1}\left(KE_{t+1},LE_{t+1},YE_{t+1}\right)}$$

*TPC* and *TEC* denote the change in technical progress and the change in technical efficiency, respectively, TFP=TPC×TEC. Since factor substitution includes the substitution of capital and labor for energy, it can still be further decomposed into the capital substitution effect (denoted as *KEC*) and labor substitution effect (denoted as *LEC*) in the global DEA framework. To avoid the uncertainty of the fixed factor reference, the geometric mean under the two-period consideration was chosen to respectively measure the capital substitution effect and labor substitution effect in this paper, and we have:

(6) $$KEC_{t}^{t+1}=\sqrt{\frac{{\bar{YE}}_{t+1}\left(KE_{t+1},LE_{t+1}\right)}{{\bar{YE}}_{t+1}\left(KE_{t},LE_{t+1}\right)}\times \frac{{\bar{YE}}_{t}\left(KE_{t+1},LE_{t}\right)}{{\bar{YE}}_{t}\left(KE_{t},LE_{t}\right)}}$$

(7) $$LEC_{t}^{t+1}=\sqrt{\frac{{\bar{YE}}_{t+1}\left(KE_{t+1},LE_{t+1}\right)}{{\bar{YE}}_{t+1}\left(KE_{t+1},LE_{t}\right)}\times \frac{{\bar{YE}}_{t}\left(KE_{t},LE_{t+1}\right)}{{\bar{YE}}_{t}\left(KE_{t},LE_{t}\right)}}$$

According to the nature of the metafrontier DEA model, production units with the same combination of factor input have the same output projection in the frontier, i.e., ${\bar{YE}}_{t}\left(KE_{t+1},LE_{t}\right)\;={\bar{YE}}_{t+1}\left(KE_{t+1},LE_{t}\right)$ and ${\bar{YE}}_{t}\left(KE_{t},LE_{t+1}\right)\;={\bar{YE}}_{t+1}\left(KE_{t},LE_{t+1}\right)$. The product of the two equals the total effect of factor substitution (*XEC)* and is easily verifiable. Carbon productivity growth is mainly driven by the factor substitution effect and the total-factor productivity. The former includes low-carbon energy substitution, capital energy substitution, and labor energy substitution, and the latter contains technical progress and efficiency improvement. Therefore, by plugging the decomposition equations of total-factor productivity and factor substitution effect into Formula (3), the decomposition equation of the quintuple growth drivers of carbon productivity can be obtained as below:

(8) $$CPC_{t}^{t+1}=ESC_{t}^{t+1}\times TPC_{t}^{t+1}\times TEC_{t}^{t+1}\times KEC_{t}^{t+1}\times LEC_{t}^{t+1}$$

It is easy to see that the change in carbon productivity is mainly driven by the energy structure effect, technological progress change, technical efficiency improvement, capital substitution effect, and labor substitution effect. Based on the above decomposition framework, growth accounting for carbon productivity changes in a country or region can be performed to assess the pattern of temporal changes dominated by the contributions of different factors.

### 3.2. β-Convergence Model of Carbon Productivity

Convergence models derived from economic growth theory can examine whether economic variables in different regions have normal and other steady states, i.e., whether lagging areas can catch up with the trends in developed areas. Common steady-state convergence is often referred to as absolute β-convergence. In contrast, different steady-state convergence is conditional β-convergence when considering the impact of differences in resource endowments and technological conditions between regions on steady-state equilibrium. We use the Mankiw–Romer–Weil (MRW) convergence model [64] as the basis, and first construct the following carbon productivity convergence model:

(9) $$\ln\left(\frac{CP_{i,t}}{CP_{i,t-1}}\right)\;=\;\alpha +\beta \ln\left(CP_{i,t-1}\right)\;+\;\varphi \ln\left(S_{i,t}\right)\;+\;\varepsilon_{i,t},\varepsilon_{it}∼N\left(0,\sigma^{2}\right)$$

where $\ln\left(CP_{i,t}/CP_{i,t-1}\right)\;=\ln\left(CPC_{i,t}\right)$ denotes the i growth rate of regional carbon productivity in two periods, and there is a log-transformation $CPC_{i,t}$ relationship with that in the decomposition model above. $\ln\left(CP_{i,t-1}\right)$ denotes the logarithm i of the initial carbon productivity level of the region, then $\ln\left(S_{i}\right)\;$ is the logarithm of the effective depreciation rate of capital in the corresponding region. α is a constant term, and the random ε, error assignment, follows a normal distribution. When β, the coefficient, is less than 0, it indicates that the carbon productivity in less-developed regions grows faster than that in developed areas, and there is a convergence trend during the study period. When the differences in regional endowment factors are φ=0 not considered, i.e., Equation (9) examines β the absolute convergence, otherwise, β is the conditional convergence.

Based on the proof of the MRW model, β it is clear that the parameters are defined by an exponential decay function, i.e.,

(10) $$\beta =e^{-vt}-1$$

In Equation (10), v  is the rate of convergence and t is the period interval examined. If the parameter takes the values −1<β<0  falling in the gap, this implies that carbon productivity can converge directly to a steady state without oscillation. Thus, according to the speed of convergence implied of β by the parameter, we can easily calculate the regression results for $v=-\ln\left(1+\beta \right)/t$.

The traditional literature ignores the spatial dependence of carbon productivity, leading to bias in estimation convergence models. The resource endowments and market conditions of neighboring provinces are generally more similar, factor flows and technological cooperation between regions will be adequate, and carbon productivity exhibits significant spatial clustering characteristics. Therefore, it is necessary to adopt a spatial panel model to revise the traditional convergence model, and we start from a spatial Durbin model (SDM) of panel data.

(11) $$\begin{array}{c} ln\left(CPC_{t}\right)\;=\;\alpha +\beta ln\left(CP_{t-1}\right)\;+\;\varphi ln\left(S_{t}\right)\;+\;\rho Wln\left(CPC_{t-1}\right)\\ +\gamma Wln\left(CP_{t-1}\right)\;+\;\theta Wln\left(S_{t}\right)\;+\;\varepsilon_{t} \end{array}$$

For the convenience of writing, the subscripts of the variables in Equation (11) are omitted to represent them in matrix and vector form. Among these, the W matrix is used to describe the dependence between regions, and the commonly used spatial adjacency weight matrix (Hainan is adjacent to Guangdong) was chosen here.  W, the product with the relative variables, represents the spatial lag term, and the other variables are defined the same as in Equation (9). It is easy to see that the SDM simultaneously examines the spatial association and mutual influence of carbon productivity and effective investment rate between regions. When the spatial lag parameter γ sum θ is zero, the SDM model degenerates to a spatial lag model (SLM), which means that the growth of carbon productivity comes only from the spatial spillover effect of the dependent variable. In addition, the unidentifiable spatial effects can be further degraded to a spatial error model (SEM) by attributing them to random error shocks if all spatial lag terms are insignificant, i.e.,

(12) $$\begin{array}{c} ln\left(CPC_{t}\right)\;=\;\alpha +\beta ln\left(CP_{t-1}\right)\;+\;\varphi ln\left(S_{t}\right)\;+\;\mu_{t}\\ \mu_{t}=\lambda W\mu_{t}+\varepsilon_{t}\;\;\;\;\;\;\;\;\;\;\;\;\;\;\;\;\;\;\;\;\;\;\;\;\; \end{array}$$

In Equation (12), $\mu_{t}\;$ is the random error term for the presence of random spatial effects, and $\varepsilon_{t}\;$ is the same, subject to the normal distribution.

Further, the spatial convergence mechanism of carbon productivity can be further tested by combining the power decomposition Equation (8) and the spatial convergence Equation (11). Substituting $\ln\left(ESC\right)$, $\ln\left(KEC\right)$, $\ln\left(LEC\right)$, $\ln\left(TPC\right)$, and $\ln\left(TEC\right)$, these denote the energy structure effect, capital substitution effect, labor substitution effect, technical progress effect, and technical efficiency improvement, respectively. The corresponding growth rate forms are obtained by taking the logarithm according to the decomposition equation. Replacing in Equation (9), $\ln\left(CPC\right)$ as the spatial convergence equation with the five major driving factors as the explanatory variables is further set. As a result, the set of joint cubic equations based on the decomposition model extension constitutes a more rigorous fully mediated effects model, and the following relationship exists between the explanatory variables of the different convergence equations β, as well as the regression coefficients, i.e.,

(13) $$\ln\left(CPC\right)\;=\ln\left(ESC\right)\;+\;\ln\left(KEC\right)\;+\;\ln\left(LEC\right)\;+\;\ln\left(TPC\right)\;+\;\ln\left(TEC\right)$$

(14) $$\beta_{CPC}=\beta_{ESC}+\beta_{KEC}+\beta_{LEC}+\beta_{TPC}+\beta_{TEC}$$

Thus, by examining the magnitude and direction of the parameters of each driver around the $\ln\left(CP_{i,t-1}\right)$, the initial level of carbon productivity can be used to assess the specific mechanisms by which carbon productivity convergence is achieved. In order to provide the accuracy of parameter estimation, quasi-maximum likelihood (QML) estimation is used for the spatial convergence model [65].

### 3.3. Variable Selection and Data Description

According to the guidelines developed by the United Nations Intergovernmental Panel on Climate Change (IPCC), the reference method accounts for fossil energy production sites. Still, many primary energy sources do not enter final consumption, resulting in severe risk of overestimation [66], so this paper chooses those fossil energy carbon emissions accounted for by the sectoral method. Based on data availability and consistency, 30 provinces, autonomous regions, and cities in China, with the exceptions of Tibet and Hong Kong, Macao, and Taiwan, were selected for the study during 1997–2017, with output as GDP, energy as total consumption, and labor as total social employees. Capital was physical capital stock and was estimated using the perpetual inventory method, which was extended to 2017 following the classical literature [67]. The underlying data are obtained from the China Statistical Yearbook, China Energy Statistical Yearbook, provincial statistical yearbooks, and the China Carbon Accounting Database (CEADs) for previous years. Considering that fossil energy is not fully converted into CO_2_, the direct use of CO_2_ emission coefficients is likely to cause estimation bias, so we used uniform carbon equivalents and reconstructed them according to provincial CO_2_ emission inventories; GDP and physical capital stock were adjusted to compare prices in 2000, and individual missing data were completed by linear interpolation.

Regarding economic growth theory, the logarithm of the ratio of capital investment rate to effective depreciation rate (g+δ) was chosen as the control variable in this paper, and $\ln S_{k}$ and $\ln S_{h}$ were logarithms of the effective investment rate of physical capital and the effective investment rate of human capital, respectively. The physical capital investment rate was measured as the share of GDP’s total fixed capital formation. The human capital investment rate used the share of the population with a high school education or above in the 15–19 age group as a proxy variable [64], g as the GDP growth rate, and  δ still used the results measured in the classical literature [67]. Corresponding data were obtained from the China Statistical Yearbook and the China Human Capital Index Report database (CHLR) for previous years. The main variables and their measures are summarized in Table 1.

## 4. Empirical Results and Discussion

### 4.1. Accounting for Carbon Productivity Growth

**Figure 1:**
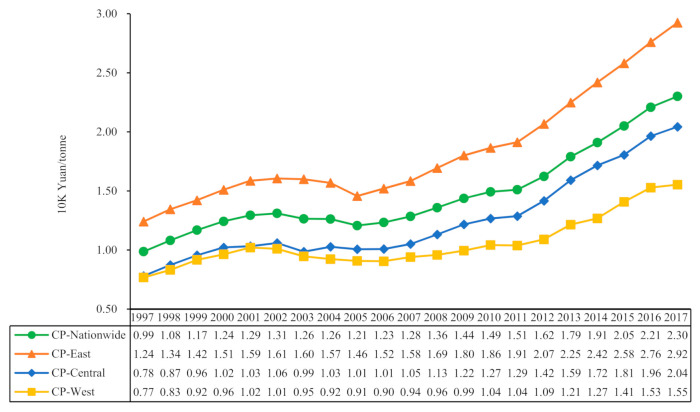
Temporal trends in carbon productivity for the country as a whole and the East, Central, and West regions.

Based on the actual GDP and carbon equivalent emissions of the sampled provinces, we first calculated the carbon productivity of the whole country and the three regions of East, West, and Central China by summing the provinces (see Figure 1). Overall, the national carbon productivity level showed a continuous positive growth trend, increasing from CNY 0.99 K/ton in 1997 to CNY 2.30 K/ton in 2017, with an average annual growth rate of 4.7%. China’s carbon productivity experienced a brief decline during 2002–2005, before the growth rate became faster, mainly due to China’s policy of energy-intensity-constrained emission reduction targets for the provinces in 2006. The implementation of the intensity reduction policy accelerated the growth rate of carbon productivity in each province, so the curve rose more significantly afterward. The carbon productivity of the three major regions of East, Central, and West has kept rising in tandem with the whole country, with 1.24, 0.78, and CNY 0.77 K/ton in 1997 and increasing to CNY 2.92, 2.04, and 1.55 K/ton in 2017, respectively. The Central region (5.3%) had the fastest average annual growth rate, while the East region (4.8%) was slightly above the national average, and the West region (4.2%) had the lowest. It is also easy to see from Figure 1 that the carbon productivity gap between the East and Central regions tended to narrow year by year. Still, the gap between the West and the other two regions significantly widened, and further statistical tests are needed to verify whether the regional carbon productivity in the country is converging or diverging. In addition, the pattern of temporal changes in carbon productivity does not show a clear linear trend, either nationally or in the East, Central, and West regions. It seems to indicate a flatter N-shaped relationship considering the previous short-term decline, but this is all inconsistent with the EKC hypothesis based on multi-country data validated in the literature [21,22,23]. The U-shaped relationship revealed by the panel data may not apply to a specific country or region [27]. Nevertheless, both curves are well above the linear trend with a goodness-of-fit of more than 0.9, which provides evidence of a nonlinear pattern of carbon productivity changes, and hypothesis 1 cannot be rejected. To focus on the highlights, we do not explore further the applicability of more EKC curve-fitting methods but turn to the analysis of the characteristics of carbon productivity changes and regional differences due to the drivers.

**Figure 2:**
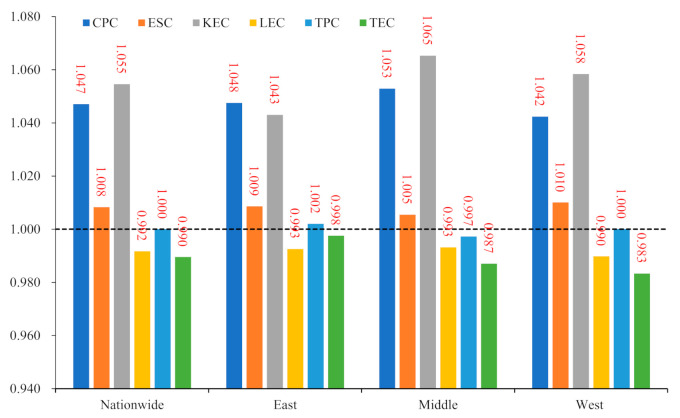
Annual average levels of carbon productivity drivers for the country as a whole and the East, Central, and West (1997–2017).

Based on the previous decomposition framework, we calculated the contribution of carbon productivity drivers for each province for each calendar year. The annual average changes in carbon productivity and its drivers were calculated here for the whole country and the East and West, using the GDP of each province as the weight, and are plotted in Figure 2. On average, national carbon productivity was mainly driven by the capital substitution effect (5.5%), followed by the energy structure effect (0.8%), with technological progress showing no significant positive or negative impact, and the labor substitution effect (−0.8%) and technical efficiency improvement (−1.0%) having an average suppressive effect, and these empirical findings are consistent with Hypothesis 2. Carbon productivity in the three regions was still dominated by capital-substitution episodic growth, with the capital substitution effect far outweighing other drivers, with the highest in the Central (6.5%), the second-highest in the West (5.8%), and the lowest in the East (4.3%). The labor substitution effect showed different degrees of deterioration, with the most severe decline in the western region. The energy structure effects in the East, Central, and West all showed different degrees of positive driving effect, with average annual growth rates of 0.8, 0.9, and 1.0 percentage points, respectively, and the speed of low-carbon energy adjustment in the three regions was not very different. The driving effect of TFP was not fully reflected in either the central or western regions, and the driving impact of technological progress on sustainable growth was more significant in the East. Hypothesis 2 is further supported by empirical evidence at the regional level.

Table 2 reports the average growth effects of carbon productivity and its drivers by region from 1997 to 2017, accounted for by the above power decomposition model. As can be seen, there were large regional differences in the absolute levels of carbon productivity in China, with Fujian being the highest and Shanxi the lowest in 1997, at CNY 2.61 and 0.36 K/ton, respectively; in 2017, Beijing ranked first with an absolute level of 6.70 and was 18.36 times higher than last place Ningxia. In terms of the average annual growth rate, except for Ningxia, which was harmful, all other regions achieved different degrees of positive growth, with Beijing leading the way with an average annual growth rate of 8.0%. So, what caused the significant regional differences in China’s carbon productivity? This can be further answered by examining the contribution of the five drivers.

The factor substitution effect was the primary determinant of regional carbon productivity growth, within which capital substitution plays a dominant role, which provides provincial-level evidence for Hypothesis 2. The results in Table 2 show that the capital substitution effect showed positive growth in all regions. In contrast, the labor substitution effect decreased to different degrees. The absolute value of the one-time contribution was much larger than that of the latter, thus making the combined impact of factor substitution significantly positive, consistent with the parametric method’s decomposition results in the literature [68]. The growth trajectories of the capital–energy ratio and labor–energy ratio in all regions during the sample period fully coincide with their corresponding substitution effects. This result also reflects the national trend of industrial restructuring characterized by factor mobility, i.e., labor-intensive industries are gradually being replaced by energy-intensive industries. Capital-intensive industries are replacing energy-intensive sectors more and more intensely. Substituting low-carbon energy for high-carbon energy can significantly suppress cumulative carbon emissions. Still, the difference in preference for energy-intensive technologies makes the polarization between regions prominent [69]. Among them, 22 areas such as Beijing and Zhejiang showed positive energy structure effects, while 8 regions such as Ningxia and Xinjiang showed negative growth. Constrained by the national “coal-based” energy endowment, the energy structure adjustment decarbonization effect in most areas was much smaller than the capital substitution effect. The contribution of technological progress and technical efficiency to carbon productivity growth was relatively low, and the total factor productivity in most regions was smaller than the capital substitution effect. This indicates that China’s provincial carbon productivity growth is generally characterized as capital substitution-driven. In contrast, total factor productivity-based organic growth dynamics are significantly insufficient, with more than half of the regions showing varying degrees of deterioration in both technical progress and technical efficiency. Among them, Shanghai was in the leading position in technological advancement. Chongqing had the most significant improvement in technical efficiency, driving the average annual growth rate of carbon productivity by 1.75 and 1.43 percentage points, respectively. In terms of interprovincial differences, the coefficient of variation (CV) of the annual average growth rate of carbon productivity was 1.96%, with the CVs of energy structure, capital substitution, labor substitution, technological progress, and technical efficiency being 1.14%, 1.46%, 0.33%, 0.72%, and 1.20%, respectively. This indicates that the difference in provincial carbon productivity growth was mainly attributed to capital substitution, followed by technical efficiency and energy structure, with a relatively small effect of technological progress and labor substitution, and Hypothesis 3 is confirmed.

### 4.2. Convergence of Carbon Productivity

#### 4.2.1. Absolute β-Convergence Estimates

We first examined the absolute convergence estimates without adding any control variables. To compare the applicability and robustness of different models, we started with least-squares estimation (OLS) for mixed data and reported the estimation results for the panel fixed effects (FE) model, panel SLM, SEM, and SDM, in turn, as shown in Table 3. The Hausman test results show that the critical probability *p*-values for all four-panel models were significant at least at the 5% statistical level, indicating that the use of individual fixed effects models was more appropriate. In addition, from the LR test results of the spatial panel models, the *p*-values were much less than 1%, indicating that the panel SDM cannot be degraded to SLM or SEM, and it was more appropriate to use SDM for testing the convergence of carbon productivity.

From the test results of absolute β-convergence, the OLS estimation results were significantly biased. At the same time, the coefficients of the other four panel models were all significant at least at the 10% statistical level, verifying the absolute β-convergence phenomenon, and Hypothesis 4 is partially confirmed. The FE estimation results obtained a convergence rate of 3.13%, ignoring that the spatial effect would produce a significant bias in the convergence estimation of carbon productivity. The SLM, SEM, and SDM estimations, which sequentially introduced the spatial lag term, obtained a convergence rate of 3.83%, 7.19%, and 22.21%, respectively, showing a significant increasing trend and gradually obtaining a substantial increase in statistical significance. Since there is an inverse transformation relationship between carbon productivity and carbon intensity, our results are consistent with the convergence tests in the literature on carbon intensity in China [16]. There is an inter-provincial absolute β-convergence trend in carbon productivity. The spatial model estimates have a higher convergence speed than the nonspatial model, and Hypothesis 5 is partially confirmed.

#### 4.2.2. Conditional  β-Convergence Estimation

Next, we examined the regional conditional convergence characteristics of China’s carbon β productivity. The results are shown in Table 4. When the regression analysis is restricted to specific individuals, fixed effects are often the better choice, as can also be seen from the Hausman test results, where the different panel models all supported estimation with individual fixed effects at the 1% statistical level, at least. Similar to the absolute β-convergence test procedure, OLS still suffered from a significant estimation bias, although the sign of the β  coefficients changed from positive to negative. However, it was not supported by a further significance test. In the conditional model estimation, the improvement in the accuracy of the convergence parameter estimates was evident by considering spatial dependence. Only the dependent convergence coefficient of SLM was slightly lower than the FE results. At the same time, the other two spatial models obtained significant improvement, and the rate of conditional convergence became more prominent as the spatial effects were more fully considered, confirming Hypothesis 4. Parameter ρ was significantly positive, which further verified the spatial dependence of China’s carbon productivity growth rate, which was characterized by the spatial clustering of “neighbors as partners”. The SDM estimation results showed a significant conditional 𝛽-convergence trend of carbon productivity in China, and that the lower-carbon-productivity regions are catching up with the developed areas faster, with an average annual convergence rate of 23.19%. This shows that spatial spillover reduces the persistent technological differences among provinces, which leads to faster convergence of carbon productivity, further confirming Hypotheses 4 and 5. This finding is consistent with recent literature [16] estimates of conditional convergence. Nevertheless, our estimates were slightly higher than those in the literature for carbon intensity convergence, both from differences in estimation methods and carbon accounting, as well as from the fact that we used control variables consistent with growth theory.

In addition, conditional convergence also implies that the relative positions of steady-state growth levels between regions are difficult to eliminate automatically in the long-term, mainly determined by regional characteristic variables’ direct and spillover effects. From the estimation results of SDM, only the spatial lag coefficient of the effective investment rate of human capital passed the significance test at the 10% statistical level. In contrast, the significance of other variables was relatively low. The differences in the effects of the control variables on the five major driving factors may obscure their significance in the total convergence equation of carbon productivity, so further identification with the help of the associative convergence model is needed.

#### 4.2.3. Heterogeneity Convergence Examination

To further examine the stage convergence and club convergence characteristics of regional carbon productivity in China, we further estimated the SDM conditional β-convergence model by stage and region. The results are shown in Table 5. Equations (1) and (2) correspond to 1997–2010 and 2011–2017, respectively. This division was based on the fact that the Chinese economy entered a distinct phase of low-to-moderate growth in 2011, which may have impacted further carbon productivity. Equations (3)–(5) correspond to the sample of provinces within the three regions of East, West, and Central China, respectively. The regional division follows the traditional way, which can be obtained from the National Bureau of Statistics of China database.

The results showed that China’s carbon productivity exhibited a spatial conditional β-convergence trend within both phases. The conditional convergence speed of carbon productivity was 40.77% in 1997–2017, while the convergence speed was as high as 70.74% in 2011–2017, the latter being significantly higher than the former. The effect of the effective investment rate in the provinces themselves did not pass the significance test in terms of control variables. The spatial spillover effect only showed positive spillover of physical capital during 1997–2010, while carbon productivity and the effective investment rate in 2011–2017 all showed significant spatial spillover effects but negative spillover of physical capital. The reason for this is that, on the one hand, since the reform and opening-up, China’s economy has long been dominated by the crude growth mode of increasing capital input, and the contribution of other factors has been dramatically weakened. On the other hand, as China’s economy enters the stage of medium-to-low growth, industrial transformation and technological upgrading become the inevitable choice for sustainable growth, and high-quality development in this stage pays more attention to the role of human capital and intellectual capital. Therefore, in the second stage, the convergence of carbon productivity is further accelerated, but the technology spillover effect characterized by human capital flow is more obvious, and the role of continuous accumulation of physical capital has turned from positive to negative.

From the regional equation estimates, the coefficients β were all significantly negative at the 1% level, indicating that China’s carbon productivity has typical club convergence characteristics and growth convergence, and Hypothesis 4 is fully confirmed. Interregional factor mobility and mutual imitation accelerate the convergence of carbon productivity, with the fastest intersection in central China (33.67%), followed by western China (31.03%), and the slowest in eastern China (17.98%). It is worth noting that physical capital accumulation had a significant negative spillover effect on carbon productivity growth in the West, because it is home to several energy-rich provinces with a high density of low-tech, high-energy-consuming, and high-emission industrial types. The blind expansion of physical capital investment by mutual imitation is not conducive to sustainable local growth, but will lead to an excessive increase in energy consumption.

### 4.3. Dynamics of Convergence

#### 4.3.1. Testing the Causes of Convergence

To further investigate the spatial convergence mechanism of carbon productivity, Table 6 reports the estimated results of the convergence equations for the five major driving factors. Firstly, we examined the convergence paths exhibited by the local initial carbon productivity levels. The corresponding coefficients β estimates which were significantly negative in Equations (1) and (2), and did not pass the significance test in Equations (3)–(5), which indicates that the convergence paths of provincial carbon productivity in China were mainly determined by the convergence mechanisms of the energy structure effect and the capital substitution effect, and Hypothesis 6 is confirmed. The convergence results for technological progress and technical efficiency were not consistent with the convergence findings in the literature on total factor productivity [9]; on the one hand, this is because the environmental DEA model dealing with undesired outputs is used in the literature. On the other hand, we used the initial carbon productivity level to estimate the coefficients β with the aim of analyzing the mechanism of the effect of the change in TFP with the initial carbon productivity level on the overall convergence, which is significantly different. In addition, the coefficients ρ were significantly positive in Equations (2)–(5). This shows obvious spatial dependence characteristics in energy structure, capital substitution, labor substitution, technological progress, and technological efficiency; the mutual imitation between regions also further accelerated the overall growth rate. The spatial lag term of initial carbon productivity passed the significance test only in Equations (1), (2), and (4). The demonstration effect of carbon productivity improvement in neighboring regions on local areas will prompt them to strengthen their energy-saving and emission-reduction regulations and promote carbon productivity growth by optimizing energy structure and accelerating technological innovation. However, the hitchhiking effect can also contribute to the weakening of local government guidance in factor optimization allocation, which is detrimental to the positive substitution of capital energy. If the demonstration effect is more significant than the hitchhiking effect or the economic steering behavior of local governments tends to choose the former, then the spatial effect of the initial carbon productivity level in neighboring regions shows positive spillover; the opposite is true. The estimation results in this paper,  0.133+0.025−0.041=0.117 showed that the demonstration effect was much more significant than the free-rider effect, which is highly consistent with the spatial clustering characteristics of carbon productivity in the country in recent years.

In terms of the mechanism of influence of the effective investment rate, both physical and human capital investment rates were mainly mediated by capital–energy substitution and total factor productivity. Among these, the direct effect of the physical capital investment rate on capital substitution (0.090) was significantly positive, while the effect on technological progress (0.031) and technical efficiency (−0.105) was in the opposite direction. This finding is consistent with China’s long-standing capital-driven growth approach, where overinvestment is also bound to cause general deterioration in total factor productivity. The coefficients of physical capital investment rates in neighboring regions passed significance tests only in Equations (2) and (4), and both had negative signs, suggesting that blindly following up on expanding investment reduces capital substitution (−0.045) and technological progress (−0.042). The local human capital investment rate helps labor substitution (0.010) and technical efficiency (0.04) to obtain significant improvements. In contrast, human capital accumulation in neighboring provinces (0.058) only showed a significant positive spatial spillover to the local capital substitution effect. Nevertheless, human capital investment’s direct and spillover effects were significantly positive. In recent years, their contribution to carbon productivity growth was further strengthened, which is also highly correlated with China’s cross-regional talent mobility trait.

#### 4.3.2. Robustness Test

The paper re-estimated the joint cubic equation using the inverse weight matrix of the latitude and longitude distances to examine further the robustness of the carbon productivity spatial convergence mechanism. Table 7 reports the results of the robustness tests of the total convergence equation and the power factor convergence equation. It is easy to see that the sign and significance of the coefficients β in Equations (1) to (6) remained unchanged. The sign of the capital investment rate coefficients and the spatial lag term remained the same as the original model. Thus, it can be judged that the spatial convergence mechanism of China’s carbon productivity estimated based on SDM was relatively robust and reliable, and the test results had some applicability. In addition, it should also be seen that the differences in the spillover effects estimated by different spatial weight matrices were mainly from the influence of their exogenous settings. Since the adjacency weight matrix responds to the average effect of local spillover and the distance weight matrix responds to the average global spillover effect, there was a significant difference in the spatial transmission mechanism between the two. However, the sign of the spatial lag coefficients of the distance weight matrix remained the same as that of the adjacency weight matrix. The significance of the global spillover effect was more polarized among the dynamic factors, which also caused the significance of the spatial lag coefficients of the total convergence Equation (1) to change.

We further used data averaged every four years, i.e., 1997–2000, 2001–2004, 2005–2008, 2009–2012, and 2013–2016, and a total of $\ln\left(CP_{i,t-1}\right)$ in the above five cycles showed that the carbon productivity level in the initial year of each cycle in each province and other variables were the average value in the cycle. This eliminated the influence of economic cycles on the model estimation on the one hand and took into account the possible influence of the lag of spatial spillover effects on the other hand. The results in Table 8 show that the sign and significance of the coefficients β in the main equations did not change. The sign of the coefficients of the control variables also remained the same as the results in Table 6, which further indicates the robustness of the previous conclusion that there is a convergence trend in the estimation of carbon productivity in China using SDM.

### 4.4. Discussion of Empirical Results

This study provides preliminary evidence of a nonlinear relationship between carbon productivity and economic growth in China, indicating that the EKC hypothesis is not valid in China. A study using autoregressive distribution lag (ARDL) modeling for Arctic countries provides similar evidence, suggesting that the EKC curves revealed by panel data may not apply to a single specific country or region [27]. Evidence of decoupling of carbon productivity is rarely found in lagging or less-developed countries due to gaps in energy structure, technology level, etc. [28,30,32]. Considering the economic growth patterns, the evolution of different drivers and their regional differences may dominate the sustainable growth process of carbon productivity in China.

Distinct from the EKC approach, this study provides a body of evidence on the drivers of carbon productivity and regional convergence in China within a new framework. Capital-to-energy substitution dominates the pattern of carbon productivity growth in China as a whole, in the East, the Center, and the West, and at the provincial level. The literature on parametric method decomposition provides similar findings [68], with the difference that we do not find evidence of drivers of technological progress, as the parametric time-invariant setting results in a forced fit of technological progress. In addition, the LMDI approach provides evidence on the drivers of macrofactors such as economic activity, industrial structure [48], and energy intensity. Still, its inability to capture input-output mechanisms is frequently cited [49,51]. Nevertheless, these empirical findings are complementary, and together they warn policymakers of the importance of sustainable growth by improving factor structure and upgrading technology. Optimizing the factor structure can help green the industry, and upgrading the structure can also improve the positive impact of total green factor productivity on pollution emissions [70].

The existing literature provides evidence of convergence in carbon intensity and energy productivity [59,71]. This study adds support for convergence in sustainable growth from the Chinese experience, where the convergence in carbon productivity across provinces and municipalities comes mainly from the effect of diminishing marginal substitution of low-carbon capital for high-carbon capital. We consider technology spillovers under spatial effects [65], and we estimate faster convergence than the closed economy hypothesis since it is easier for lagging regions to access low-carbon capital and learn low-carbon technologies in an open economy. Similar empirical findings also provide evidence of convergence in carbon intensity [16]. This study also closely links the convergence analysis to the drivers and finds that carbon productivity convergence in China is mainly achieved through conjunction in the energy structure and capital substitution. Unlike the generalized carbon productivity convergence in the non-expected output perspective [9,70], our decomposition framework does not find total factor productivity convergence evidence. Nevertheless, this complementary empirical evidence shows that the spatial Solow model reasonably explains sustainable growth.

Therefore, policymakers should develop new policies to attract low-carbon-oriented extraterritorial investments and promote information exchange and technology transfer, which may provide the basis for re-establishing sustainable growth ambitions. Policymakers in lagging regions should strengthen environmental regulations to prevent increased environmental degradation due to energy-intensive and emission-intensive capital inflow [72]. From a management perspective, policies on environmentally sustainable behavior help regulate corporate social responsibility, which will ultimately positively impact a sustainable and equitable future for all, as it is widely understood, accepted, and implemented by stakeholders [73]. In addition, engaging in environmental protection and environmentally sustainable behavior can further improve corporate reputation. A good reputation is a valuable organizational resource that positively and significantly impacts customer behavior [74].

## 5. Conclusions and Implications

Carbon productivity has a richer economic connotation as a core variable of sustainable growth. Based on the global DEA model, the present paper constructed the relation between factor substitution, technical progress, technical efficiency, and carbon productivity. The present study measured the five driving forces of carbon productivity growth in 30 provinces, autonomous regions, and cities based on the data sample during 1997–2017. The work further tested and analyzed the spatial convergence mechanism of provincial carbon productivity in China using the spatial panel model.

The empirical results show that the capital substitution growth pattern generally dominates China’s provincial carbon productivity. Energy restructuring mostly has positive effects, and labor substitution effects show different degrees of deterioration. The regional differences in total factor productivity are significant, with more pronounced efficiency-based growth in the Eastern region and a general deterioration in the Central and Western areas. These empirical findings are consistent with China’s economic growth, as sloppy growth that relies on rapid capital accumulation is bound to undermine a sustainable growth path centered on carbon productivity. China’s provincial carbon productivity growth has robust spatial absolute β-convergence and spatial conditional β-convergence trends. Low-carbon-productivity regions have catch-up effects relative to high-carbon-productivity regions, and spatial spillover effects significantly accelerate regional convergence. SDM is more suitable for testing China’s carbon productivity convergence pattern. The spatial conditions of carbon productivity in China exhibit two stages of convergence. After entering the medium and low growth stage, the economic transition accelerates the rate of carbon productivity convergence. Similarly to economic convergence, China’s carbon productivity exhibits typical club convergence characteristics, with faster convergence in the Central and Western regions than in the East. While energy mix and capital substitution dominate each province’s carbon productivity growth pattern, they also determine the overall carbon productivity convergence trend. The mechanism of effective investment rate on carbon productivity growth rate is mainly mediated by capital substitution and total factor productivity. Physical capital investment has gradually shifted to an inhibitory effect, while the positive push of human capital investment is strengthening. Expanding physical capital investment can directly improve the local capital substitution effect. Still, it is gradually offset by the deterioration of efficiency caused by investment imbalance, which is more serious in terms of spatial effects. Expanding human capital investment can significantly improve local factor allocation, and factor mobility and mutual imitation across regions can significantly enhance the contribution of human capital spillovers to carbon productivity growth. China’s sustainable growth will increasingly rely on human capital accumulation in the form of technology spillovers.

The above findings have important policy implications for China in the promotion of sustainable growth, with carbon productivity at its core. Since capital energy substitution has dominant empirical evidence, policymakers should continuously optimize the design and implementation strategies, strengthen the structure and quality of capital inputs, and restrain the excessive growth of energy factors. Policymakers should impose strict restrictions on those backward industries with high energy consumption and high emissions, and control the carbon productivity decline caused by their production scale expansion. The adoption of low-energy consumption capital and the development of independent innovation capacity are the main policy instruments. Given the positive effect of human capital on carbon productivity improvement, policymakers should strengthen the guidance of human capital investment to improve the factor input structure. In economically backward inland regions, policymakers should fully consider the technology spillover effects of human capital investment to benefit local and global sustainable growth. The spatial technology spillover effect accelerates the overall convergence process. This finding provides a scientific basis for policymakers to sustain innovation support and regional cooperation in low-carbon, energy, and other environmental technologies. It enables the sustainable growth advantages of lagging provinces and cities to be fully unleashed. Inter-regional trade and technical exchanges are inseparable from supporting an excellent infrastructure and institutional system. Therefore, promoting the construction of infrastructures such as transportation and information transmission and improving the institutional mechanism of inter-regional exchange and cooperation should also attract the full attention of policymakers. In addition, the results of this study provide Chinese experiences and Chinese cases for global energy conservation and emission reduction, especially for developing countries or regions that adopt intensity reduction strategies. A formal unified constraint system, effective regulation of energy-intensive capital, and enhanced cooperation in low-carbon technologies remain valuable references for decision-making.

The above findings have important practical implications for carbon intensity reduction practices and sustainable growth programs in China and other countries. The policy implications are as follows: firstly, we should continue to deepen the structural reform on the supply side of energy, further strengthen the control of excessive accumulation of energy factors, optimize the energy consumption structure, accelerate the technological transformation of coal energy consumption, and improve energy utilization efficiency. Secondly, we should pay attention to the guiding role of fixed asset investment efficiency, strengthen the control of regional investment growth rates and spatial structure regulation, and gradually realize the reasonable and orderly substitution of capital factors for energy factors. Again, improving human capital investment, especially in economically less-developed inland areas, gives full play to the technological spillover effect of human capital investment and actively promotes the joint innovation and coordinated development of production technology, energy technology, and environmental technology. Finally, we should develop and implement differentiated regional development strategies, promote infrastructure construction such as transportation and information transmission, increase the depth and breadth of exchanges and cooperation between regions, especially between developed and less-developed regions, and further release the spatial spillover effects of factor flows and technology diffusion.

The relatively short period of the dataset provides a potential limitation of this study, while the natural process of convergence may take decades or even longer to end. Our findings may indicate initial signs of convergence in carbon productivity, and it may be easier for provinces to collaborate in future synergies in emission reduction targets. It is crucial to investigate this relationship further. In addition, our study only provides empirical evidence. It does not provide a more in-depth theoretical proof of the spatial convergence mechanism of carbon productivity, which is also missing in the existing literature, and is undoubtedly an important research topic for sustainable growth theory.

## Figures and Tables

**Table 1:** Variable descriptions and measurements.

Variable	Symbol	Unit	Description of Indicator
Carbon productivity	*CP*	CNY 10 K/ton C	The ratio of GDP to carbon equivalent emissions
Energy Productivity	YE	CNY 10 K/tce	The ratio of GDP to standard coal energy consumption
Capital energy substitution	KE	CNY 10 K/tce	The ratio of total capital input to total energy input
Labor energy substitution	LE	Person/tce	The ratio of total labor input to total energy input
Energy consumption structure	ES	tce/ton C	The ratio of total energy consumption to total carbon emissions
Effective investment rate of physical capital	Sk	/	The ratio of fixed asset investment rate to the effective depreciation rate
Effective investment rate of human capital	Sh	/	The ratio of investment rate in human capital to effective depreciation rate

**Table 2:** Annual average levels of provincial carbon productivity drivers (1997–2017).

Region	CP_1997	CP_2017	CPC	ESC	KEC	LEC	TPC	TEC
Beijing	1.426	6.704	1.080	1.015	1.054	0.999	1.015	0.996
Tianjin	0.922	3.658	1.071	1.009	1.051	0.996	1.012	1.003
Hebei	0.695	1.334	1.033	0.998	1.057	0.993	0.993	0.993
Shanxi	0.363	0.726	1.035	0.994	1.067	0.994	0.994	0.988
Neimenggu	0.442	0.810	1.031	1.005	1.067	0.985	1.000	0.976
Liaoning	0.687	1.759	1.048	1.006	1.055	0.992	0.999	0.997
Jilin	0.573	2.155	1.068	0.998	1.103	0.994	0.998	0.978
Heilongjiang	0.722	2.168	1.057	1.004	1.063	0.994	0.995	1.001
Shanghai	1.266	4.463	1.065	1.013	1.038	0.995	1.017	1.000
Jiangsu	1.347	2.891	1.039	0.999	1.051	0.989	0.997	1.004
Zhejiang	1.588	3.573	1.041	1.008	1.042	0.991	0.995	1.006
Anhui	0.809	1.956	1.045	0.996	1.063	0.996	0.988	1.004
Fujian	2.606	4.130	1.023	0.999	1.051	0.991	0.996	0.988
Jiangxi	1.207	2.330	1.033	1.002	1.057	0.994	0.990	0.992
Shandong	1.247	2.487	1.035	1.001	1.050	0.990	0.995	1.000
Henan	0.996	2.379	1.044	1.005	1.077	0.997	0.993	0.975
Hubei	0.800	2.659	1.062	1.020	1.067	0.991	0.994	0.990
Hunan	1.109	2.827	1.048	1.008	1.060	0.994	0.990	0.996
Guangdong	1.929	4.586	1.044	1.013	1.045	0.992	0.995	0.999
Guangxi	1.313	2.451	1.032	1.005	1.067	0.993	0.993	0.976
Hainan	2.247	2.912	1.013	1.000	1.016	0.990	0.999	1.008
Chongqing	0.983	3.507	1.066	1.026	1.041	0.989	0.994	1.014
Sichuan	0.976	3.353	1.064	1.016	1.060	0.995	0.990	1.004
Guizhou	0.419	1.086	1.049	1.007	1.072	0.995	0.991	0.985
Yunnan	1.093	2.463	1.041	1.010	1.069	0.995	0.992	0.978
Shaanxi	0.743	1.855	1.047	1.004	1.068	0.989	0.995	0.992
Gansu	0.607	1.447	1.044	1.002	1.071	0.997	0.988	0.989
Qinghai	0.667	1.236	1.031	1.016	1.059	0.988	1.000	0.970
Ningxia	0.498	0.365	0.985	0.960	1.068	0.989	0.999	0.971
Xinjiang	0.649	0.658	1.001	0.992	1.038	0.989	1.001	0.982
CV	51.58	55.03	1.96	1.14	1.46	0.33	0.72	1.20

Data source: this table is compiled by accounting; carbon productivity and driver changes are annual averages; and the coefficient of variation (CV) is the percentage of the ratio of standard deviation to the mean.

**Table 3:** Absolute *β*-convergence estimation results of carbon productivity.

Variables	(1) OLS	(2) FE	(3) SLM	(4) SEM	(5) SDM
*β*	0.0071 (0.01)	−0.0308 (0.02) *	−0.0376 (0.02) **	−0.0694 (0.03) **	−0.1992 (0.05) ***
W × ln*CP*					0.2307 (0.05) ***
*ρ*			0.1965 (0.05) ***		0.2209 (0.05) ***
*λ*				0.2627 (0.06) ***	
Constant	0.0397 (0.00) ***	0.0491 (0.01) ***			
Sigma2_e			0.0085 (0.00) ***	0.0084 (0.00) ***	0.0077 (0.00) ***
LR (*p*-value)			60.98 (0.000) ***	54.85 (000) ***	
Hausman (*p*-value)		7.38 (0.007) ***	6.65 (0.036) **	6.23 (0.045) **	26.57 (0.000) ***
Convergence speed	−0.71%	3.13%	3.83%	7.19%	22.21%
N	600	600	600	600	600
R2	0.0017	0.0068	0.0028	0.0068	0.0975

Note: ***, **, and * are significant at the 1%, 5%, and 10% levels, respectively; robust standard errors are in parentheses for parameters, and *p*-values are in parentheses for diagnostic tests.

**Table 4:** Conditional *β*-convergence estimation results for carbon productivity.

Variables	(1) OLS	(2) FE	(3) SLM	(4) SEM	(5) SDM
*β*	−0.0184 (0.01)	−0.1447 (0.03) ***	−0.1423 (0.03) ***	−0.1457 (0.04) ***	−0.2070 (0.05) ***
lnSk	−0.0501 (0.03) *	-0.0171 (0.04)	−0.0177 (0.04)	−0.0115 (0.04)	−0.0355 (0.04)
lnSh	0.0764 (0.02) ***	0.1277 (0.02) ***	0.1215 (0.02) ***	0.1222 (0.02) ***	−0.0083 (0.06)
W × lnCP					0.0867 (0.05)
W × lnSk					−0.0129 (0.08)
W × lnSh					0.1390 (0.08) *
*ρ*			0.1097 (0.06) *		0.1240 (0.04) ***
*λ*				0.1079 (0.05) **	
Constant	0.0327 (0.01) **	0.0045 (0.02)			
Sigma2_e			0.0078 (0.00) ***	0.0078 (0.00) ***	0.0075 (0.00) ***
LR (*p*-value)			22.03 (0.000) ***	22.63 (0.000) ***	
Hausman (*p*-value)		53.74 (0.000) ***	20.86 (0.000) ***	22.75 (0.000) ***	21.82 (0.003) ***
Convergence speed	1.86%	15.63%	15.35%	15.75%	23.19%
N	600	600	600	600	600
R2	0.0510	0.1129	0.1143	0.1127	0.1451

Note: ***, **, and * are significant at the 1%, 5%, and 10% levels, respectively; robust standard errors are in parentheses for parameters, and *p*-values are in parentheses for diagnostic tests.

**Table 5:** Estimation results of stage convergence and club convergence based on the SDM model.

Variables	(1) 1997–2010	(2) 2011–2017	(3) East	(4) Central	(5) West
*β*	−0.3348 (0.07) ***	−0.5071 (0.08) ***	−0.1646 (0.03) ***	−0.2859 (0.07) ***	−0.2668 (0.09) ***
lnSk	−0.0888 (0.06)	0.1036 (0.08)	−0.0714 (0.05)	−0.0091 (0.04)	0.0314 (0.08)
lnSh	−0.0655 (0.09)	0.0027 (0.08)	−0.0415 (0.05)	0.1231 (0.08)	0.0454 (0.11)
W × lnCP	0.0413 (0.08)	0.3125 (0.08) ***	0.0997 (0.05) **	−0.0013 (0.06)	0.2018 (0.11) *
W × lnSk	0.2342 (0.11) **	−0.3729 (0.16)**	0.2239 (0.15)	−0.0758 (0.09)	−0.2836 (0.15) *
W × lnSh	0.0995 (0.09)	0.3442 (0.09) ***	0.0636 (0.08)	0.1402 (0.10)	0.1368 (0.16)
*ρ*	0.0764 (0.05)	0.0869 (0.09)	0.1415 (0.06) **	0.0198 (0.03)	0.0771 (0.06)
Sigma2_e	0.0078 (0.00) ***	0.0037 (0.00) ***	0.0040 (0.00) ***	0.0054 (0.00) ***	0.0118 (0.00) ***
Convergence speed	40.77%	70.74%	17.98%	33.67%	31.03%
N	390	210	220	160	220
R2	0.2156	0.3377	0.2015	0.2505	0.1427

Note: ***, **, and * are significant at the 1%, 5%, and 10% levels, respectively; robust standard errors are in parentheses for parameters, and p-values are in parentheses for diagnostic tests.

**Table 6:** Tests of the convergence mechanism of carbon productivity based on SDM model.

Variables	(1) lnESC	(2) lnKEC	(3) lnLEC	(4) lnTPC	(5) lnTEC
*β*	−0.1759 (0.05) ***	−0.0371 (0.02) **	0.0031 (0.00)	−0.0022 (0.01)	0.0062 (0.01)
lnSk	−0.0440 (0.04)	0.0902 (0.03) ***	0.0006 (0.01)	0.0310 (0.01) ***	−0.1054 (0.02) ***
lnSh	−0.0423 (0.05)	−0.0244 (0.03)	0.0099 (0.01) *	0.0068 (0.01)	0.0410 (0.01) ***
W × lnCP	0.1332 (0.05) **	−0.0413 (0.02) **	−0.0006 (0.00)	0.0249 (0.01) **	−0.0147 (0.02)
W × lnSk	0.0463 (0.07)	−0.0445 (0.02) *	−0.0071 (0.01)	−0.0420 (0.01) ***	0.0267 (0.03)
W × lnSh	0.0835 (0.06)	0.0579 (0.02) ***	−0.0058 (0.01)	−0.0152 (0.01)	0.0047 (0.02)
*ρ*	0.0393 (0.04)	0.3990 (0.04) ***	0.4578 (0.04) ***	0.6185 (0.04) ***	0.2953 (0.06) ***
Sigma2_e	0.0067 (0.00) ***	0.0014 (0.00) ***	0.0001 (0.00) ***	0.0002 (0.00) ***	0.0004 (0.00) ***
Hausman (*p*-value)	19.99 (0.006) ***	12.96 (0.073) *	25.43 (0.001) ***	16.97 (0.018) **	20.77 (0.004) ***
Convergence speed	19.35%	3.78%	−0.31%	0.22%	−0.62%
N	600	600	600	600	600
R2	0.0958	0.1919	0.0233	0.1690	0.1582

Note: ***, **, and * are significant at the 1%, 5%, and 10% levels, respectively; robust standard errors are in parentheses for parameters, and p-values are in parentheses for diagnostic tests.

**Table 7:** Robustness test of the SDM model based on spatial distance weights.

Variables	(1) lnCPC	(2) lnESC	(3) lnKEC	(4) lnLEC	(5) lnTPC	(6) lnTEC
*β*	−0.2011 (0.06) ***	−0.1707 (0.05) ***	−0.0395 (0.02) **	0.0034 (0.00)	0.0017 (0.00)	0.0093 (0.02)
lnSk	−0.0284 (0.04)	−0.0312 (0.04)	0.0906 (0.03) ***	−0.0026 (0.01)	0.0214 (0.01) ***	−0.1082 (0.02) ***
lnSh	−0.0338 (0.06)	−0.0557 (0.05)	−0.0362 (0.03)	0.0079 (0.01)	0.0120 (0.01) *	0.0500 (0.02) ***
W × lnCP	0.0248 (0.06)	0.0881 (0.06)	−0.0600 (0.03) **	−0.0008 (0.01)	0.0069 (0.01)	−0.0172 (0.02)
W × lnSk	0.0007 (0.17)	−0.0237 (0.16)	0.0387 (0.07)	0.0119 (0.02)	−0.0846 (0.03) ***	0.1153 (0.05) **
W × lnSh	0.1863 (0.12)	0.1540 (0.11)	0.0408 (0.05)	−0.0133 (0.01)	0.0139 (0.02)	−0.0473 (0.03) *
*ρ*	0.2971 (0.06) ***	0.0687 (0.06)	0.5345 (0.09) ***	0.6544 (0.06) ***	0.7713 (0.04) ***	0.4870 (0.07) ***
Sigma2_e	0.0073 (0.00) ***	0.0067 (0.00) ***	0.0014 (0.00) ***	0.0001 (0.00) ***	0.0002 (0.00) ***	0.0004 (0.00) ***
Hausman (*p*-value)	37.41 (0.000) ***	31.34 (0.000) ***	17.82 (0.013) **	20.71 (0.004) ***	10.91 (0.143)	24.00 (0.001)
Convergence speed	22.45%	18.72%	4.03%	−0.34%	−0.17%	−0.93%
N	600	600	600	600	600	600
R2	0.1630	0.0948	0.2590	0.0164	0.3402	0.1918

Note: ***, **, and * are significant at the 1%, 5%, and 10% levels, respectively; robust standard errors are in parentheses for parameters, and p-values are in parentheses for diagnostic tests.

**Table 8:** Robustness test of the SDM model based on average data.

Variables	(1) lnCPC	(2) lnESC	(3) lnKEC	(4) lnLEC	(5) lnTPC	(6) lnTEC
*β*	−0.1085 (0.03) ***	−0.0922 (0.02) ***	−0.0300 (0.01) **	0.0059 (0.00) **	−0.0046 (0.01)	0.0154 (0.02)
lnSk	−0.0449 (0.05)	−0.0415 (0.04)	0.0529 (0.03)	−0.0023 (0.01)	0.0379 (0.01) ***	−0.0872 (0.02) ***
lnSh	0.0052 (0.05)	−0.0641 (0.04) *	0.0007 (0.03)	0.0160 (0.01) ***	0.0029 (0.01)	0.0571 (0.02) ***
W × lnCP	−0.0068 (0.04)	0.0718 (0.03) **	−0.0801 (0.02) ***	−0.0104 (0.01)	0.0289 (0.01) **	−0.0443 (0.02) *
W × lnSk	0.0229 (0.08)	0.1035 (0.0623) *	−0.0416 (0.03)	−0.0147 (0.01)	−0.0333 (0.02) **	−0.0163 (0.04)
W × lnSh	0.0948 (0.04) **	0.0557 (0.04)	0.0668 (0.03) **	−0.0026 (0.01)	−0.0200 (0.01) **	0.0146 (0.03)
*ρ*	0.3150 (0.09) ***	0.1595 (0.08) *	0.3731 (0.07) ***	0.5743 (0.06) ***	0.6854 (0.04) ***	0.1789 (0.09) *
Sigma2_e	0.0011 (0.00) ***	0.0008 (0.00) ***	0.0006 (0.00) ***	0.0000 (0.00) ***	0.0001 (0.00) ***	0.0003 (0.00) ***
Hausman (*p*-value)	15.44 (0.031) **	17.04 (0.017) **	13.08 (0.040) *	21.64 (0.003) ***	12.22 (0.09) *	16.67 (0.020) **
Convergence speed	11.48%	9.67%	3.05%	−0.59%	0.46%	−1.53%
N	150	150	150	150	150	150
R2	0.3955	0.1962	0.4276	0.0935	0.3657	0.2024

Note: ***, **, and * are significant at the 1%, 5%, and 10% levels, respectively; robust standard errors are in parentheses for parameters, and p-values are in parentheses for diagnostic tests.

## Data Availability

Not applicable.
